# Differential Associations of Cognitive Function, Frailty, and Comorbidity Burden with Visual Field Sensitivity and Reliability in Glaucoma

**DOI:** 10.3390/biomedicines14071513

**Published:** 2026-07-05

**Authors:** Yuya Kato, Mayumi Furue, Chisako Ida, Hinako Ohtani, Kana Murakami, Mizuki Koike, Keigo Takagi, Yuto Yoshida, Kazunobu Sugihara, Masaki Tanito

**Affiliations:** Department of Ophthalmology, Shimane University Faculty of Medicine, Izumo 693-8501, Japan

**Keywords:** glaucoma, visual field, comprehensive geriatric assessment, Mini-Cog, frailty, Age-Adjusted Charlson Comorbidity Index, visual field reliability, cognitive impairment, false-negative responses, false-positive responses

## Abstract

**Background/Objectives**: Cognitive impairment, frailty, and systemic comorbidity burden are common in elderly patients with glaucoma and may influence both visual field (VF) performance and glaucoma severity. This study investigated the associations of comprehensive geriatric assessment (CGA) parameters, including Mini-Cog, G8, and Age-Adjusted Charlson Comorbidity Index (ACCI), with VF sensitivity and VF reliability indices in glaucoma patients. **Methods**: This retrospective cross-sectional study included 1125 eyes of 622 glaucoma patients who underwent Humphrey VF testing and CGA at a tertiary referral center. Associations between CGA parameters and VF indices, including mean deviation (MD), pattern standard deviation (PSD), foveal sensitivity, fixation loss rate (FL), false-negative rate (FN), and false-positive rate (FP), were evaluated. Generalized linear mixed models were used to assess independent associations after adjustment for demographic, systemic, and ocular covariates. **Results**: In univariate analyses, lower Mini-Cog and G8 scores and higher ACCI scores were associated with several VF sensitivity and reliability indices. After multivariable adjustment, ACCI remained independently associated with lower MD (estimate = −0.52, *p* = 0.004), higher PSD (estimate = 0.27, *p* = 0.04), and lower foveal sensitivity (estimate = −0.36, *p* = 0.01). Lower G8 scores and higher ACCI scores were independently associated with increased FN rates, whereas higher G8 scores were associated with increased FP rates. **Conclusions**: Systemic comorbidity burden, assessed using ACCI, was independently associated with both glaucomatous functional impairment and selected VF reliability indices. Frailty, assessed using G8, was associated with VF reliability but not VF sensitivity. Although cognitive function measured by Mini-Cog was associated with VF parameters in univariate analyses, these associations were not retained after multivariable adjustment. Consideration of systemic health status and geriatric vulnerability may improve interpretation of VF results in patients with glaucoma.

## 1. Introduction

Glaucoma is a chronic progressive optic neuropathy and one of the leading causes of irreversible blindness worldwide [1]. Visual field (VF) testing remains an essential tool for the diagnosis, staging, and longitudinal monitoring of glaucoma because functional deterioration is directly reflected in VF sensitivity measurements [2]. Standard automated perimetry, particularly the Humphrey Visual Field Analyzer, is widely used in routine glaucoma practice and clinical research. However, VF testing is a psychophysical examination that requires sustained attention, adequate cognitive processing, stable fixation, and appropriate physical endurance [3,4]. Consequently, test performance and reliability may be influenced not only by glaucomatous damage itself but also by patient-related systemic and geriatric factors.

The aging of the global population has increased the number of elderly glaucoma patients in daily clinical practice [5]. Aging is frequently accompanied by cognitive decline, frailty, and multiple systemic comorbidities [6], all of which may potentially affect VF examination performance. Cognitive dysfunction may impair task understanding, response consistency, and concentration during perimetry, whereas frailty and systemic disease burden may reduce physical endurance and attentional capacity [7,8,9]. In addition, systemic comorbidities may contribute directly to glaucomatous damage through vascular, metabolic, inflammatory, or neurodegenerative mechanisms [10,11]. These factors could therefore influence not only VF reliability parameters, including fixation losses (FL), false-negative (FN), and false-positive (FP) responses, but also VF sensitivity indices reflecting the severity of glaucomatous functional impairment. Despite these observations, it remains unclear whether geriatric vulnerability and systemic health status are associated only with the quality of VF testing or also with the severity of glaucomatous functional impairment itself. Furthermore, the independent contributions of cognitive function, frailty, and systemic comorbidity to VF sensitivity and VF reliability after adjustment for demographic and ocular confounding factors have not been fully elucidated.

Comprehensive geriatric assessment (CGA) is an established multidimensional approach for evaluating elderly individuals by assessing cognitive function, frailty, physical condition, and systemic comorbidity burden [12,13]. Among available CGA-related tools, the Mini-Cog is a brief cognitive screening test designed to detect possible cognitive impairment [14,15], the G8 screening tool is commonly used to assess frailty and geriatric vulnerability [16], and the Age-Adjusted Charlson Comorbidity Index (ACCI) quantifies systemic comorbidity burden while incorporating the influence of aging [17,18]. These instruments are relatively simple and practical for use in clinical settings and may provide useful information regarding both glaucoma severity and the ability of elderly patients to reliably undergo VF testing. These instruments are relatively simple and practical for use in routine clinical settings because they can be completed rapidly without specialized equipment. Importantly, they evaluate complementary domains of geriatric health, including cognition (Mini-Cog), frailty (G8), and systemic comorbidity burden (ACCI), thereby providing a comprehensive yet feasible assessment of factors that may influence both glaucomatous functional impairment and the reliability of VF testing in older adults.

Several previous studies have suggested associations between cognitive impairment and reduced VF test reliability or glaucomatous functional deterioration [7,9,19]. However, most studies have focused primarily on cognition alone [7,9,19], and relatively few investigations have simultaneously evaluated cognitive status, frailty, systemic comorbidity burden, VF sensitivity parameters, and VF reliability indices within a large glaucoma population. Furthermore, the independent effects of these geriatric parameters after adjustment for demographic and ocular confounding factors remain unclear.

Therefore, the present study aimed to investigate the associations between CGA parameters, including Mini-Cog, G8, and ACCI scores, and VF sensitivity indices as well as VF reliability indices in patients with glaucoma. In addition, we evaluated whether these associations remained significant after adjustment for demographic and ocular background variables using generalized linear mixed models. Particular attention was given to determining whether cognitive status, frailty, or systemic comorbidity burden independently influenced glaucomatous functional severity and the reliability of VF testing.

## 2. Materials and Methods

### 2.1. Subjects

This retrospective investigation was conducted in accordance with the tenets of the Declaration of Helsinki and received approval from the Institutional Review Board of Shimane University Hospital (approval No. 20200228-2; originally approved on 26 March 2020 and revised on 27 April 2026). The requirement for written informed consent was waived by the IRB because an opt-out approach was adopted; details of the study were publicly disclosed at the participating institution, allowing eligible individuals to decline participation. The study population comprised 1125 eyes from 622 consecutive Japanese patients (340 men and 282 women) who attended the glaucoma clinic at Shimane University Hospital between June 2023 and June 2025. Eligible subjects had at least one eye diagnosed with glaucoma, including primary open-angle glaucoma (PG), primary angle-closure disease (AC), or exfoliation glaucoma (EG), had undergone visual field testing using the Humphrey central 30-2 program, and had completed a comprehensive geriatric assessment (CGA) consisting of the Mini-Cog, G8, and Charlson Comorbidity Index (CCI). Patients with ocular diseases other than glaucoma and cataract that would cause vision loss were excluded. Patients with neurological disorders, including dementia, stroke, and Parkinson’s disease, were not excluded because these conditions contribute to cognitive function, frailty, and systemic comorbidity burden, which were evaluated using the comprehensive geriatric assessment instruments in the present study. In our routine clinical practice, both glaucoma medication adherence assessments and CGA evaluations are generally performed during the initial visit or as part of preoperative examinations. Visual field (VF) examinations were not excluded based on reliability indices because the study aimed to investigate the association between CGA parameters and VF reliability. Excluding unreliable VFs could have introduced selection bias and obscured this relationship.

### 2.2. Data Collection

Clinical records were retrospectively reviewed to obtain both subject-level and eye-level data. Subject-level variables included the results of the three CGA tests, age at the time of CGA assessment, sex, and the presence or absence of hypertension (HT) and diabetes mellitus (DM). Eye-level variables included lens status, glaucoma subtype, best-corrected visual acuity (BCVA), spherical equivalent refractive error (SERE), intraocular pressure (IOP) measured, axial length (AL), and VF parameters. The VF examination performed closest to the timing of the CGA assessment was selected for analysis. In most cases, the CGA assessment and VF examination were performed on the same day. The recorded highest intraocular pressure (IOP) value was used for analysis, whereas all other ophthalmic parameters were obtained from examinations performed closest to the timing of the CGA assessments. Histories of HT and DM were identified from self-reported medical information and records of systemic medication use. Glaucoma subtype classification was performed independently for each eye by one investigator (M.T.) on the basis of comprehensive ophthalmic findings, including IOP measurement, slit-lamp biomicroscopy, fundus examination, gonioscopy, and VF assessment. Diagnostic categorization followed the criteria described in the Japan Glaucoma Society Guidelines for Glaucoma, 5th edition [20]. Normal-tension glaucoma was categorized as PG, whereas eyes with exfoliation syndrome were categorized as EG. Primary angle-closure glaucoma, primary angle closure, and primary angle-closure suspect were collectively categorized as AC. Because glaucoma subtype assignment was conducted on an eye-specific basis, the two eyes of a single subject were not necessarily classified into the same subtype. Decimal BCVA values were converted to logarithm of the minimum angle of resolution (LogMAR) units. Visual acuity levels corresponding to counting fingers, hand motions, light perception, and no light perception were converted to decimal values of 0.0025, 0.002, 0.0016, and 0.0013, respectively [21]. SERE was measured using an autorefractometer (TonoRef III, Nidek, Gamagori, Japan). IOP measurements were obtained with a Goldmann applanation tonometer. AL was determined by optical biometry (OA-2000; Tomey Corporation, Nagoya, Japan). Visual field examinations were performed using the Humphrey Field Analyzer Central 30-2 program (Carl Zeiss Meditec, Dublin, CA, USA) with the Swedish Interactive Threshold Algorithm (SITA)-standard strategy. Extracted VF parameters included mean deviation (MD), pattern standard deviation (PSD), foveal sensitivity, fixation loss rate (FL), false-negative rate (FN), and false-positive rate (FP). Among the study variables, only foveal sensitivity contained missing values (216 eyes), because this parameter is turned off by default during Humphrey visual field testing. No imputation was performed, and analyses involving foveal sensitivity were conducted using the available data.

### 2.3. CGA Tests

Mini-Cog: Cognitive function was screened using the Mini-Cog instrument [14], which consists of a three-item delayed recall task combined with a clock-drawing exercise designed to evaluate memory and executive functioning. The total score ranges from 0 to 5. Scores between 0 and 2 were regarded as suggestive of cognitive decline, whereas scores of 3 to 5 were interpreted as cognitively preserved. The test can usually be completed within a few minutes.

G8 Screening Tool: Frailty-related status and geriatric vulnerability were assessed using the G8 screening questionnaire [16]. This tool includes eight domains covering decreased food intake over the previous three months, unintentional weight reduction, impaired mobility, neuropsychological disturbances, low body mass index (BMI ≤ 21), polypharmacy (three or more medications daily), self-perceived health condition, and advanced age (≥85 years). Scores range from 0 to 17, with values of 14 or below generally considered indicative of frailty or the need for comprehensive geriatric evaluation.

Age-Adjusted Charlson Comorbidity Index (ACCI): Systemic comorbidity burden was assessed using the Charlson Comorbidity Index (CCI) [18]. The CCI allocates weighted scores to various chronic medical conditions, including cardiovascular, cerebrovascular, respiratory, hepatic, renal, metabolic, and malignant diseases, as well as dementia and acquired immunodeficiency syndrome (AIDS). Larger cumulative scores reflect more severe systemic illness and a greater predicted risk of mortality. To incorporate the influence of aging, age-related weighting was added according to the Age-Adjusted Charlson Comorbidity Index (ACCI): 1 point for 41–50 years, 2 points for 51–60 years, 3 points for 61–70 years, 4 points for 71–80 years, and 5 points for ≥81 years [17]. This modification enables a more comprehensive estimation of health risk in older individuals by considering both comorbidity burden and age-related vulnerability.

The G8 and CCI assessments were conducted by ophthalmologists, while the Mini-Cog examination was administered by ophthalmic nurses during patient instruction sessions for glaucoma eye-drop use. All assessments were performed face-to-face in an interview-based manner.

### 2.4. Statistical Analysis

Continuous variables are presented as mean ± standard deviation (SD) together with 95% confidence intervals (CIs), whereas categorical variables are summarized as numbers and percentages. Relationships between CGA scores and continuous background variables including CGA scores were examined using linear regression analyses. For categorical variables, comparisons between two groups were performed using the *t*-test, while a comparison among three groups was analyzed using one-way analysis of variance (ANOVA) followed by Tukey’s honestly significant difference (HSD) post hoc test. The associations between CGA scores and VF parameters, including MD, PSD, foveal sensitivity, FL, FN, and FP, were evaluated using generalized linear mixed models (GLMMs) with Gaussian distribution and an identity link function. Although FL, FN, and FP are bounded percentage variables, they were analyzed as continuous outcomes because the primary objective of the present study was to estimate associations between CGA parameters and visual field reliability indices while accounting for within-subject correlation. Because both eyes from the same subject could be included in the analysis, subject identification number was incorporated as a random intercept to account for intra-subject correlation. Three separate multivariable models were constructed for each outcome variable. In each model, only one CGA parameter (Mini-Cog, G8, or ACCI) was entered as the primary explanatory variable to avoid collinearity among the CGA measures. Age, sex, lens status, glaucoma subtype, BCVA, SERE, IOP, AL, HT, and DM were included as covariates in the Mini-Cog-adjusted model. Because age is incorporated into both G8 and ACCI, age was excluded from the G8-adjusted and ACCI-adjusted models to avoid overadjustment and potential collinearity. All statistical analyses were conducted using JMP Student Edition version 19.1.1 (SAS Institute, Cary, NC, USA). A *p* value of less than 0.05 was considered statistically significant.

## 3. Results

Table 1 summarizes the demographic and ophthalmic characteristics of the study population at both the subject and eye levels. A total of 622 subjects (1125 eyes) were included. The mean age was 69.3 ± 12.6 years, and 340 subjects (54.7%) were male. Hypertension and diabetes mellitus were present in 56.6% and 14.6% of subjects, respectively. The mean scores for the Mini-Cog, G8, and ACCI were 4.3 ± 1.0, 14.3 ± 1.8, and 3.7 ± 1.6, respectively.

Among the analyzed eyes, 57.7% were phakic and 42.2% had intraocular lenses. PG was the most frequent glaucoma subtype (72.9%), followed by EG (20.4%) and AC (6.8%). Mean BCVA was 0.13 ± 0.34 LogMAR, mean SERE was −2.6 ± 3.3 diopters, and the highest recorded IOP averaged 22.9 ± 9.1 mmHg. Mean AL was 25.1 ± 1.8 mm. Regarding VF indices, the mean MD, PSD, and foveal sensitivity values were −7.2 ± 7.1 dB, 7.5 ± 5.0 dB, and 33.4 ± 6.0 dB, respectively. The mean FL, FN, and FP rates, which represent reliability indices of VF testing, were 13.4 ± 17.4%, 5.3 ± 7.3%, and 4.0 ± 6.8%, respectively.

Table 2 summarizes the associations between CGA scores and continuous clinical parameters. Increasing age was significantly associated with lower Mini-Cog and G8 scores and higher ACCI scores (all *p* < 0.0001). Worse BCVA was significantly associated with lower Mini-Cog scores and higher ACCI scores, whereas no significant association was observed with the G8 score. More myopic SERE and longer AL were significantly associated with higher Mini-Cog and G8 scores and lower ACCI scores (all *p* < 0.001). No significant relationships were observed between IOP and any CGA score. Among visual field parameters, lower foveal sensitivity was significantly associated with lower Mini-Cog scores (*p* = 0.01) and higher ACCI scores (*p* < 0.0001). Higher FN rates, one of the reliability indices of visual field testing, were significantly associated with lower Mini-Cog and G8 scores and higher ACCI scores. Higher FL rates were significantly associated with higher ACCI scores, whereas FP rates were not significantly associated with any CGA parameter. MD was significantly associated only with ACCI, while PSD showed no significant correlations with CGA scores.

Table 3 presents the associations between CGA scores and categorical variables. Female subjects had significantly lower G8 scores than male subjects (*p* < 0.0001), whereas Mini-Cog and ACCI scores did not differ significantly by sex. Eyes with intraocular lenses showed significantly lower G8 scores and higher ACCI scores compared with phakic eyes (*p* = 0.0003 and *p* < 0.0001, respectively), while Mini-Cog scores were comparable between the two groups.

Table 4 shows the associations between glaucoma subtypes and CGA scores. Significant differences among glaucoma subtypes were observed for all three CGA parameters. Mini-Cog scores differed significantly across glaucoma subtypes (overall *p* = 0.0006), with both EG and AC eyes showing significantly lower scores than PG eyes (*p* = 0.04 and *p* = 0.003, respectively). No significant difference in Mini-Cog score was observed between EG and AC eyes. G8 scores also differed significantly among subtypes (overall *p* = 0.0001). EG eyes demonstrated significantly lower G8 scores compared with PG eyes (*p* < 0.0001), whereas AC eyes did not significantly differ from either PG or EG eyes. For ACCI, significant differences were likewise detected among glaucoma subtypes (overall *p* < 0.0001). Both EG and AC eyes had significantly higher ACCI scores than PG eyes (both *p* = 0.002 or smaller), and EG eyes also showed significantly higher ACCI scores than AC eyes (*p* = 0.04).

Table 5, Table 6 and Table 7 summarize the multivariate analyses evaluating the associations between CGA scores and major VF parameters after adjustment for demographic and ocular background variables using generalized linear mixed models. For MD (Table 5), ACCI showed a significant negative association with MD (estimate = −0.52, *p* = 0.004), indicating that greater systemic comorbidity burden was independently associated with more severe VF loss. In contrast, Mini-Cog and G8 scores were not significantly associated with MD after multivariable adjustment. Among the covariates, pseudophakia, worse BCVA, higher IOP, and the EG subtype were significantly associated with MD. Specifically, the EG subtype was associated with higher MD values compared with PG eyes. More myopic SERE was associated with better MD values. In the G8-adjusted model, HT was independently associated with lower MD values. For PSD (Table 6), ACCI showed a significant positive association with PSD (estimate = 0.27, *p* = 0.04), whereas Mini-Cog and G8 scores were not significantly associated with PSD after adjustment for covariates. Among the background variables, pseudophakia, worse BCVA, more hyperopic refractive status, and the EG subtype were significantly associated with PSD values. Specifically, the EG subtype was associated with lower PSD values compared with PG eyes. No significant associations were observed for IOP, AL, HT, or DM. For foveal sensitivity (Table 7), ACCI showed a significant negative association with foveal sensitivity (estimate = −0.36, *p* = 0.01), whereas Mini-Cog and G8 scores were not significantly associated with foveal sensitivity. Worse BCVA showed the strongest association with reduced foveal sensitivity in all models (all *p* < 0.0001). Pseudophakia was also significantly associated with lower foveal sensitivity in all adjusted models. No significant associations were identified for glaucoma subtype, IOP, AL, HT, or DM in the adjusted analyses.

Table 8, Table 9 and Table 10 summarize the multivariate analyses evaluating associations between CGA scores and VF reliability indices. For FL (Table 8), none of the CGA parameters showed significant independent associations after adjustment for demographic and ocular covariates. Worse BCVA was consistently associated with higher FL values in all models (all *p* ≤ 0.0007). In the G8-adjusted and ACCI-adjusted models, DM was independently associated with higher FL values. No significant associations were observed for glaucoma subtype, IOP, AL, HT, lens status, or sex. For FN (Table 9), both G8 and ACCI showed significant independent associations with FN. Lower G8 scores were associated with higher FN rates (estimate = −0.31, *p* = 0.03), whereas higher ACCI scores were associated with higher FN rates (estimate = 0.57, *p* = 0.003). Mini-Cog was not significantly associated with FN. Among the covariates, older age, worse BCVA, and higher IOP were independently associated with increased FN rates. No significant associations were identified for glaucoma subtype, lens status, SERE, AL, HT, DM, or sex. For FP (Table 10), higher G8 scores were significantly associated with increased FP rates (estimate = 0.28, *p* = 0.04), whereas Mini-Cog and ACCI were not significantly associated with FP. Female sex was consistently associated with higher FP rates across all models (all *p* ≤ 0.0003). AC eyes demonstrated significantly lower FP rates compared with PG eyes. In addition, HT was significantly associated with higher FP rates in the G8-adjusted and ACCI-adjusted models. Worse BCVA was associated with lower FP rates in the Mini-Cog-adjusted model. No significant associations were observed for EG subtype, lens status, SERE, IOP, AL, or DM.

## 4. Discussion

The present study investigated the potential influence of comprehensive geriatric assessment (CGA) parameters, including Mini-Cog, G8, and ACCI, on VF sensitivity and VF reliability indices in patients with glaucoma. To our knowledge, few studies have simultaneously evaluated cognitive status, frailty, systemic comorbidity burden, VF sensitivity parameters, and VF reliability indices within a large glaucoma cohort. The major findings of this study were as follows. First, univariate analyses demonstrated significant associations between CGA parameters and several VF indices, particularly foveal sensitivity, FL, and FN. Second, after multivariable adjustment, ACCI remained independently associated with MD, PSD, and foveal sensitivity, whereas Mini-Cog and G8 were not independently associated with major VF sensitivity parameters. Third, G8 and ACCI remained independently associated with selected VF reliability indices, particularly FN, while G8 was additionally associated with FP. Fourth, several ocular and demographic factors, including BCVA, IOP, glaucoma subtype, and sex, demonstrated independent associations with VF sensitivity and reliability measures.

In the univariate analyses, poorer cognitive or geriatric status tended to be associated with worse VF performance. Lower Mini-Cog scores were associated with lower foveal sensitivity and higher FN rates, while lower G8 scores were also associated with increased FN rates. Higher ACCI scores were associated with worse MD, lower foveal sensitivity, and increased FL and FN rates. These findings are clinically plausible because cognitive decline, frailty, and systemic disease burden may reduce attention, concentration, fixation stability, and test endurance during automated perimetry. Previous studies have similarly reported associations between cognitive impairment and reduced VF reliability, increased VF variability, and decreased VF sensitivity in glaucoma patients [7,9,19]. VF testing requires sustained psychophysical responses over several minutes, and elderly patients with cognitive or systemic vulnerability may therefore demonstrate unstable responses or reduced test reliability [7,9].

After adjustment for demographic and ocular confounders using generalized linear mixed models, ACCI remained independently associated with multiple indices of VF damage, including lower MD, higher PSD, and lower foveal sensitivity. These findings suggest that systemic comorbidity burden may have a stronger relationship with glaucomatous functional impairment than cognitive screening or frailty screening alone. Recent studies have also suggested that frailty and systemic health status may be associated with glaucoma risk and visual field progression [22,23,24]. Systemic diseases incorporated within ACCI, including cardiovascular, cerebrovascular, renal, and metabolic disorders, may influence ocular perfusion, vascular autoregulation, oxidative stress, inflammatory pathways, and overall physiological reserve, all of which have been implicated in glaucoma pathophysiology and progression [25,26]. In contrast, Mini-Cog and G8 were not independently associated with VF sensitivity measures after adjustment, suggesting that their apparent associations in univariate analyses were largely explained by shared demographic and clinical factors. Because the present study was cross-sectional, the observed association between ACCI and VF sensitivity cannot distinguish whether a greater systemic comorbidity burden contributes to true glaucomatous functional impairment through vascular or neurodegenerative mechanisms, or whether it partly reflects reduced test performance resulting from fatigue or other patient-related factors during perimetry. Longitudinal studies incorporating both structural and functional outcomes will be required to clarify these mechanisms. Although the regression coefficient for ACCI was approximately −0.5 dB per point, the cumulative effect across the observed range of ACCI scores may be clinically meaningful. For example, a difference of 3–5 ACCI points corresponds to an estimated difference of approximately 1.5–2.5 dB in MD after adjustment, which may influence the clinical interpretation of disease severity.

Interestingly, the relationships between CGA parameters and VF reliability indices differed from those observed for VF sensitivity. In our previous study, abnormal Mini-Cog scores were independently associated with higher FN and FP rates among glaucoma patients, suggesting that cognitive impairment adversely affects VF reliability [9]. In another study from our institution, abnormal Mini-Cog scores were associated with thinner inner macular structures, worse MD values, and higher FN and FP rates, indicating associations between cognitive impairment and both structural and functional glaucoma severity [19]. In contrast, Mini-Cog was not independently associated with either VF sensitivity or VF reliability indices in the present multivariable analyses. Several methodological differences may account for these discrepancies. First, both previous studies evaluated cognitive function primarily as a categorical variable based on abnormal Mini-Cog scores, whereas the present study analyzed Mini-Cog scores as a continuous variable. Second, the present models included a broader range of demographic, systemic, and ocular covariates, including glaucoma subtype, refractive status, axial length, lens status, HT, and DM. These factors may have attenuated the apparent associations between cognitive function and VF outcomes observed in earlier analyses. Taken together, these findings suggest that cognitive impairment remains associated with glaucoma severity and VF reliability at the univariate level, but these associations were attenuated after adjustment for demographic, systemic, and ocular covariates, whereas systemic comorbidity burden remained independently associated with several VF outcomes.

With respect to VF reliability indices, neither Mini-Cog, G8, nor ACCI was independently associated with FL. However, lower G8 scores and higher ACCI scores were independently associated with higher FN rates, while higher G8 scores were associated with increased FP rates. The association between lower G8 scores and higher FN rates appears clinically plausible because frail individuals may experience reduced concentration, increased fatigue, or diminished response consistency during perimetric testing [6]. In contrast, the association between higher G8 scores and increased FP rates was unexpected. One possible explanation is that individuals with better overall physical and cognitive function may be more likely to respond aggressively during threshold testing, resulting in an increased tendency toward false-positive responses. Alternatively, this finding may reflect residual confounding or chance associations arising from the relatively small effect size. Because few previous studies have specifically evaluated the relationship between frailty and FP responses, this exploratory finding should be interpreted cautiously and requires confirmation in independent cohorts. Taken together, these contrasting associations may reflect differences in response behavior during perimetric testing according to frailty status. Frail individuals may exhibit reduced responsiveness because of fatigue, slower reaction times, or diminished attention, resulting in a tendency toward higher FN and lower FP responses. Conversely, less frail individuals may demonstrate greater responsiveness and a lower threshold for responding, which could contribute to relatively higher FP rates. Although this interpretation is biologically plausible, it remains speculative and warrants confirmation in future studies. FN responses are also known to increase with greater glaucomatous VF damage. Therefore, disease severity may have partially contributed to the observed associations. Nevertheless, the independent associations of lower G8 scores and higher ACCI scores with FN rates after multivariable adjustment suggest that geriatric vulnerability and systemic comorbidity may also contribute to VF reliability. Taken together, these findings suggest that geriatric vulnerability and systemic comorbidity burden may influence response consistency during perimetry even when their associations with glaucomatous damage itself are limited [7,9]. Frail individuals may be more susceptible to fatigue-related response failures [6], whereas variations in attention, response strategy, or test engagement may contribute to alterations in FP performance [9]. Furthermore, FN and other reliability indices have long been recognized as being influenced not only by glaucomatous damage but also by patient performance during perimetric testing [3]. Collectively, these findings suggest that geriatric vulnerability may affect the quality of VF testing as well as the interpretation of VF reliability indices [3,7].

Several other findings are noteworthy. Worse BCVA consistently demonstrated strong associations with multiple VF reliability indices and with reduced foveal sensitivity, emphasizing the importance of central visual function in perimetric performance [3]. Higher IOP was independently associated with increased FN rates and worse MD values. Female sex was associated with higher FP rates across all models, although the underlying mechanism remains uncertain. HT and DM also demonstrated independent associations with selected VF reliability indices. HT was associated with lower MD values and higher FP rates in some adjusted models, whereas DM was associated with higher FL rates. HT may influence VF performance through alterations in ocular perfusion and vascular autoregulation, both of which have been implicated in glaucoma pathophysiology [26,27]. In contrast, DM has been associated with both glaucoma and cognitive dysfunction, reduced attention, and impaired psychomotor performance, which could potentially affect fixation stability during perimetric testing [28,29]. Nevertheless, because the observed associations were not entirely consistent across models, these findings should be interpreted cautiously and warrant further investigation. AC eyes demonstrated lower FP rates compared with PG eyes. An unexpected finding was that EG eyes showed higher MD values and lower PSD values than PG eyes after multivariable adjustment. This result appears inconsistent with the univariate comparisons, in which EG eyes exhibited poorer CGA profiles, including lower Mini-Cog and G8 scores and higher ACCI scores. Importantly, as described in the Section 2, the EG group included not only eyes with exfoliation glaucoma but also eyes with exfoliation syndrome. Consequently, this category may have included relatively mild or preperimetric eyes, potentially contributing to the unexpectedly higher MD and lower PSD values after adjustment. Second, adjustment for age, systemic comorbidities, refractive status, lens status, and other covariates may have altered the apparent relationship between glaucoma subtype and VF severity. In addition, glaucoma subtype was classified on an eye-specific basis, whereas CGA parameters were assessed at the subject level. Finally, the PG group included normal-tension glaucoma, which is highly prevalent in Japanese populations and may have influenced the distribution of VF severity [30]. Because the present study was not specifically designed to compare VF severity among glaucoma subtypes, these findings should be interpreted cautiously and require confirmation in future studies.

From a clinical perspective, the present findings suggest that patients with greater frailty or systemic comorbidity may benefit from individualized perimetric testing strategies. For example, allowing additional rest during testing, minimizing patient fatigue, or considering shorter perimetric protocols when clinically appropriate may help improve test reliability in vulnerable older adults. In addition, clinicians should interpret VF reliability indices with particular caution in patients with high geriatric vulnerability. Several limitations should also be considered. First, the retrospective cross-sectional design precludes causal inference. Second, the study population consisted exclusively of Japanese patients at a single tertiary referral center, potentially limiting generalizability. Third, Mini-Cog and G8 are screening tools and do not establish formal diagnoses of dementia or frailty [15,16]. Fourth, detailed educational, psychological, and socioeconomic factors that may influence VF performance were not assessed. Finally, longitudinal analyses were not performed; therefore, the effects of geriatric vulnerability on future VF progression or long-term VF reliability remain uncertain. Despite these limitations, the present study has several strengths. A relatively large number of subjects and eyes were included, and multiple CGA domains were simultaneously evaluated. In addition, the use of generalized linear mixed models allowed adjustment for both-eye inclusion and multiple confounding variables. Furthermore, this study evaluated not only VF sensitivity indices but also VF reliability indices, which are highly relevant in real-world glaucoma practice, particularly in elderly patients.

## 5. Conclusions

In conclusion, after adjustment for demographic and ocular confounders, ACCI remained independently associated with MD, PSD, and foveal sensitivity, suggesting an association between systemic comorbidity burden and glaucomatous functional impairment. In addition, lower G8 scores and higher ACCI scores were associated with increased FN rates, while higher G8 scores were associated with increased FP rates, suggesting that frailty and systemic comorbidity burden may influence VF reliability. Although Mini-Cog demonstrated significant associations with several VF parameters in univariate analyses, these associations were no longer significant after multivariable adjustment. These findings highlight the importance of considering systemic health status and geriatric vulnerability when interpreting both VF severity and VF reliability in patients with glaucoma. Further longitudinal studies are required to determine whether these associations reflect true glaucomatous progression or differences in visual field test performance related to patient factors.

## Figures and Tables

**Table 1:** Demographic Data Based on Subjects and Eyes.

Parameters	N or Mean ± SD	% or 95%CI
**Subjects**	622	
Age, year	69.3 ± 12.6	68.3, 70.3
Sex		
male	340	54.7
female	282	45.3
HT		
yes	352	56.6
no	270	43.4
DM		
yes	91	14.6
no	531	85.4
Mini-Cog	4.3 ± 1.0	4.3, 4.4
G8	14.3 ± 1.8	14.1, 14.4
ACCI	3.7 ± 1.6	3.6, 3.8
**Eyes**	1125	
Lens status		
Phakia	650	57.7
IOL	475	42.2
The type of glaucoma		
PG	820	72.9
EG	229	20.4
AC	76	6.8
BCVA, LogMAR	0.13 ± 0.34	0.11, 0.15
SERE, D	−2.6 ± 3.3	−2.8, −2.4
IOP, mmHg	22.9 ± 9.1	22.5, 23.5
AL, mm	25.1 ± 1.8	25.0, 25.1
MD, dB	−7.2 ± 7.1	−7.6, −6.8
PSD, dB	7.5 ± 5.0	7.2, 7.8
Fovea, dB	33.4 ± 6.0	33.0, 33.8
FL, %	13.4 ± 17.4	12.3, 14.4
FN, %	5.3 ± 7.3	4.8, 5.7
FP, %	4.0 ± 6.8	3.6, 4.4

SD, standard deviation; 95%CI, 95% confidence interval; HT, hypertension; DM, diabetes mellitus; Mini-Cog, Mini-Cognitive Assessment Instrument; G8, G8 geriatric screening tool; ACCI, Age-Adjusted Charlson Comorbidity Index; IOL, intraocular lens; PG, primary open-angle glaucoma; EG, exfoliation glaucoma; AC, angle-closure glaucoma; BCVA, Best-Corrected Visual Acuity; LogMAR, logarithm of the minimum angle of resolution; SERE, spherical equivalent refractive error; IOP, intraocular pressure; AL, axial length; MD, visual field mean deviation; PSD, visual field pattern standard deviation; FL, fixation losses; FN, false negative; FP, false positive; Fovea, foveal sensitivity.

**Table 2:** Associations between CGA scores and continuous variables.

Parameters	Mini-Cog			G8			ACCI		
	Estimate	95%CI	*p*-Value	Estimate	95%CI	*p*-Value	Estimate	95%CI	*p*-Value
Age, year	−3.48	−4.22, −2.74	<0.0001 **	−1.42	−1.83, −1.01	<0.0001 **	6.83	6.58, 7.09	<0.0001 **
BCVA, LogMAR	−0.04	−0.06, −0.02	0.0001 **	−0.01	−0.02, 0.00	0.07	0.04	0.03, 0.05	<0.0001 **
SERE, D	−0.42	−0.62, −0.22	<0.0001 **	−0.21	−0.32, −0.10	0.0002 **	0.90	0.78, 1.01	<0.0001 **
IOP, mmHg	0.27	−0.28, 0.83	0.33	0.00	−0.30,0.30	0.99	−0.10	−0.44, 0.24	0.57
AL, mm	0.35	0.25, 0.46	<0.0001 **	0.20	0.14, 0.26	<0.0001	−0.46	−0.52, −0.39	<0.0001 **
MD, dB	0.18	−0.25, 0.61	0.41	0.13	−0.10, 0.36	0.28	−0.57	−0.83, −0.30	<0.0001 **
PSD, dB	0.12	−0.18, 0.42	0.45	0.06	−0.10, 0.23	0.44	0.17	−0.02, 0.35	0.08
Fovea, dB	0.57	0.16, 0.98	0.01 *	0.18	−0.04, 0.39	0.11	−0.58	−0.82, −0.34	<0.0001 **
FL, %	−0.84	−1.89, 0.22	0.12	−0.01	−0.58, 0.59	0.97	0.77	0.12,1.43	0.02 *
FN, %	−0.52	−0.96, −0.07	0.02 *	−0.40	−0.64, −0.15	0.002 **	0.69	0.41, 0.96	<0.0001 **
FP, %	0.25	−0.17, 0.67	0.24	0.21	−0.02, 0.43	0.07	−0.15	−0.41, 0.11	0.26

*p*-values are calculated by linear regression analysis. The * and ** denote significant levels of 5% (*p* < 0.05) and 1% (*p* < 0.01), respectively. CGA, comprehensive geriatric assessment; 95%CI, 95% confidence interval; Mini-Cog, Mini-Cognitive Assessment Instrument; G8, G8 geriatric screening tool; ACCI, Age-Adjusted Charlson Comorbidity Index; BCVA, Best-Corrected Visual Acuity; LogMAR, logarithm of the minimum angle of resolution; SERE, spherical equivalent refractive error; IOP, intraocular pressure; AL, axial length; MD, visual field mean deviation; PSD, visual field pattern standard deviation; Fovea, foveal sensitivity; FL, fixation losses; FN, false negative; FP, false positive.

**Table 3:** Associations between CGA scores and categorical variables.

Parameters	Mini-Cog			G8			ACCI		
**Sex**	**male**	**female**	***p*** **-value**	**male**	**female**	***p*** **-value**	**male**	**female**	***p*** **-value**
Mean ± SD	4.3 ± 0.9	4.4 ± 1.0	0.52	14.5 ± 1.7	14.1 ± 1.8	<0.0001 **	3.7 ± 1.6	3.7 ± 1.5	0.80
95%CI	4.3, 4.4	4.3, 4.4		14.4, 14.7	13.9, 14.3		3.5, 3.8	3.6, 3.8	
**Lens status**	**phakic**	**IOL**	***p*** **-value**	**phakic**	**IOL**	***p*** **-value**	**phakic**	**IOL**	***p*** **-value**
Mean ± SD	4.3 ± 1.0	4.3 ± 1.0	0.99	14.5 ± 1.7	14.1 ± 1.8	0.0003 **	3.3 ± 1.6	4.2 ± 1.3	<0.0001 **
95%CI	4.3, 4.4	4.3, 4.4		14.4, 14.6	13.9, 14.3		3.1, 3.4	4.1, 4.4	

*p*-values are calculated by *t*-test. The ** denotes significant level of 1% (*p* < 0.01). CGA, comprehensive geriatric assessment; Mini-Cog, Mini-Cognitive Assessment Instrument; G8, G8 geriatric screening tool; ACCI, Age-Adjusted Charlson Comorbidity Index; SD, standard deviation; 95%CI, 95% confidence interval; IOL, intraocular lens.

**Table 4:** Association between CGA scores and Glaucoma Subtypes.

Parameters	Mini-Cog				G8				ACCI			
	PG	EG	AC	*p*-Value ^a^	PG	EG	AC	*p*-Value ^a^	PG	EG	AC	*p*-Value ^a^
Mean ± SD	4.4 ± 0.9	4.2 ± 1.0	4.0 ± 1.3	0.0006 **	14.5 ± 1.8	13.9 ± 1.9	14.3 ± 1.5	0.0001 **	3.4 ± 1.5	4.5 ± 1.4	4.0 ± 1.4	<0.0001 **
95%CI	4.3, 4.5	4.1, 4.4	3.7, 4.3		14.3, 14.6	13.7, 14.1	13.9, 14.6		3.3, 3.5	4.3, 4.7	3.7, 4.4	
*p*-value, vs. PG ^b^	-	0.04 *	0.003 **		-	<0.0001 **	0.69		-	<0.0001 **	0.002 **	
*p*-value, vs. EG ^b^	-	-	0.25		-	-	0.23		-	-	0.04 *	

^a^ *p*-values are calculated by one-way ANOVA. ^b^ *p*-values are calculated by Tukey’s HSD. The * and ** denote significant levels of 5% (*p* < 0.05) and 1% (*p* < 0.01), respectively. CGA, comprehensive geriatric assessment; Mini-Cog, Mini-Cognitive Assessment Instrument; G8, G8 geriatric screening tool; ACCI, Age-Adjusted Charlson Comorbidity Index; PG, primary open-angle glaucoma; EG, exfoliation glaucoma; AC, angle-closure glaucoma; SD, standard deviation; 95%CI, 95% confidence interval.

**Table 5:** Multivariate analysis of possible associations between MD and demographic parameters.

Parameters	Mini-Cog			G8			ACCI		
	Estimate	95%CI	*p*-Value	Estimate	95%CI	*p*-Value	Estimate	95%CI	*p*-Value
Age, /y	−0.04	−0.09, 0.01	0.10	-	-	-	-	-	-
Sex, F/M	0.71	−0.23, 1.66	0.14	0.72	−0.22, 1.67	0.13	0.62	−0.32, 1.56	0.19
Lens, IOL/phakic	−2.50	−3.54, −1.46	<0.0001 **	−2.74	−3.73, −1.76	<0.0001 **	−2.39	−3.41, −1.38	<0.0001 **
Gla, AC/PG	−1.64	−3.56, 0.27	0.09	−1.67	−3.58, 0.24	0.09	−1.61	−3.51, 0.29	0.10
Gla, EG/PG	1.55	0.38, 2.71	0.01 *	1.36	0.22, 2.50	0.02 *	1.62	0.47, 2.78	0.006 **
BCVA, /LogMAR	−4.80	−6.03, −3.57	<0.0001 **	−4.99	−6.20, −3.79	<0.0001 **	−4.70	−5.92, −3.49	<0.0001 **
SERE, /D	0.25	0.06, 0.43	0.01 *	0.23	0.05, 0.42	0.01 *	0.26	0.08, 0.45	0.005 **
IOP, /mmHg	−0.09	−0.14, −0.04	0.0002 **	−0.08	−0.13, −0.04	0.0006 **	−0.09	−0.14, −0.05	0.0001 **
AL, /mm	−0.13	−0.50, 0.24	0.49	−0.06	−0.42, 0.30	0.74	−0.16	−0.52, 0.20	0.38
HT, yes/no	0.89	−0.08, 1.85	0.07	−1.07	−2.01, −0.13	0.03 *	−0.85	−1.80, 0.10	0.08
DM, yes/no	−0.30	−1.61, 1.02	0.66	0.23	−1.08, 1.54	0.73	0.31	−1.00, 1.61	0.64
Mini-Cog	0.02	−0.48, 0.51	0.95	-	-	-	-	-	-
G8	-	-	-	0.10	−0.16, 0.36	0.45	-	-	-
ACCI	-	-	-	-	-	-	−0.52	−0.88, −0.17	0.004 **

*p*-values are calculated by Generalized Linear Mixed Model. The * and ** denote significant levels of 5% (*p* < 0.05) and 1% (*p* < 0.01), respectively. Mini-Cog, Mini-Cognitive Assessment Instrument; G8, G8 geriatric screening tool; ACCI, Age-Adjusted Charlson Comorbidity Index; 95%CI, 95% confidence interval; IOL, intraocular lens (pseudophakic); Gla, glaucoma; AC, angle-closure; EG, exfoliation; PG, primary open-angle; BCVA, Best-Corrected Visual Acuity; LogMAR, logarithm of the minimum angle of resolution; IOP, intraocular pressure; AL, axial length; HT, hypertension; DM, diabetes mellitus.

**Table 6:** Multivariate analysis of possible associations between PSD and demographic parameters.

Parameters	Mini-Cog			G8			ACCI		
	Estimate	95%CI	*p*-Value	Estimate	95%CI	*p*-Value	Estimate	95%CI	*p*-Value
Age, /y	0.03	−0.00, 0.07	0.07	-	-	-	-	-	-
Sex, F/M	−0.28	−0.94, 0.39	0.41	−0.23	−0.90, 0.43	0.49	−0.21	−0.87, 0.46	0.54
Lens, IOL/phakic	1.51	0.76, 2.26	<0.0001 **	1.76	1.05, 2.47	<0.0001 **	1.53	0.80, 2.26	<0.0001 **
Gla, AC/PG	0.43	−0.92, 1.78	0.53	0.39	−0.96, 1.74	0.57	0.38	−0.97, 1.72	0.58
Gla, EG/PG	−1.77	−2.61, −0.92	<0.0001 **	−1.59	−2.42, −0.77	0.0002 **	−1.76	−2.59, −0.93	<0.0001 **
BCVA, /LogMAR	1.37	0.45, 2.28	0.004 **	1.51	0.61, 2.40	0.0010 **	1.32	0.42, 2.23	0.004 **
SERE, /D	−0.15	−0.28, −0.02	0.03 *	−0.14	−0.27, −0.01	0.04 *	−0.15	−0.29, −0.02	0.03 *
IOP, /mmHg	0.03	−0.01, 0.06	0.14	0.02	−0.02, 0.05	0.28	0.03	−0.01, 0.06	0.15
AL, /mm	0.13	−0.13, 0.39	0.32	0.06	−0.19, 0.32	0.62	0.14	−0.12, 0.40	0.29
HT, yes/no	−0.36	−1.04, 0.32	0.30	0.5	−0.17, 1.16	0.14	0.39	−0.28, 1.06	0.25
DM, yes/no	0.27	−0.66, 1.19	0.57	−0.2	−1.12, 0.73	0.68	−0.26	−1.19, 0.66	0.57
Mini-Cog	0.15	−0.20, 0.50	0.40	-	-	-	-	-	-
G8	-	-	-	0.05	−0.14, 0.23	0.61	-	-	-
ACCI	-	-	-	-	-	-	0.27	0.01, 0.52	0.04 *

*p*-values are calculated by Generalized Linear Mixed Model. The * and ** denote significant levels of 5% (*p* < 0.05) and 1% (*p* < 0.01), respectively. Mini-Cog, Mini-Cognitive Assessment Instrument; G8, G8 geriatric screening tool; ACCI, Age-Adjusted Charlson Comorbidity Index; 95%CI, 95% confidence interval; IOL, intraocular lens (pseudophakic); Gla, glaucoma; AC, angle-closure; EG, exfoliation; PG, primary open-angle; BCVA, Best-Corrected Visual Acuity; LogMAR, logarithm of the minimum angle of resolution; IOP, intraocular pressure; AL, axial length; HT, hypertension; DM, diabetes mellitus.

**Table 7:** Multivariate analysis of possible associations between Foveal sensitivity and demographic parameters.

Parameters	Mini-Cog			G8			ACCI		
	Estimate	95%CI	*p*-Value	Estimate	95%CI	*p*-Value	Estimate	95%CI	*p*-Value
Age, /y	−0.04	−0.08, 0.00	0.06	-	-	-	-	-	-
Sex, F/M	−0.08	−0.85, 0.69	0.84	−0.09	−0.86, 0.68	0.81	−0.16	−0.93, 0.61	0.68
Lens, IOL/phakic	−0.88	−1.73, −0.02	0.04 *	−1.14	−1.95, −0.32	0.006 **	−0.86	−1.70, −0.02	0.04 *
Gla, AC/PG	−1.05	−2.71, 0.61	0.21	−1.03	−2.69, 0.63	0.22	−1.03	−2.68, 0.62	0.22
Gla, EG/PG	0.35	−0.59, 1.29	0.46	0.14	−0.78, 1.06	0.77	0.34	−0.59, 1.27	0.48
BCVA, /LogMAR	−9.63	−10.71, −8.55	<0.0001 **	−9.86	−10.92, −8.81	<0.0001 **	−9.64	−10.71, −8.58	<0.0001 **
SERE, /D	0.08	−0.079, 0.24	0.32	0.07	−0.09, 0.22	0.41	0.09	−0.07, 0.24	0.29
IOP, /mmHg	−0.01	−0.05, 0.032	0.70	0	−0.04, 0.04	0.90	−0.01	−0.05, 0.03	0.74
AL, /mm	−0.20	−0.51, 0.12	0.21	−0.11	−0.42, 0.19	0.46	−0.21	−0.52, 0.10	0.19
HT, yes/no	−0.18	−0.97, 0.62	0.66	−0.02	−0.79, 0.75	0.96	0.14	−0.63, 0.92	0.72
DM, yes/no	0.69	−0.40, 1.78	0.21	−0.75	−1.84, 0.34	0.18	−0.69	−1.78, 0.39	0.21
Mini-Cog	0.07	−0.34, 0.48	0.75	-	-	-	-	-	-
G8	-	-	-	0.03	−0.18, 0.24	0.77	-	-	-
ACCI	-	-	-	-	-	-	−0.36	−0.64, −0.08	0.01 *

*p*-values are calculated by Generalized Linear Mixed Model. The * and ** denote significant levels of 5% (*p* < 0.05) and 1% (*p* < 0.01), respectively. Mini-Cog, Mini-Cognitive Assessment Instrument; G8, G8 geriatric screening tool; ACCI, Age-Adjusted Charlson Comorbidity Index; 95%CI, 95% confidence interval; IOL, intraocular lens (pseudophakic); Gla, glaucoma; AC, angle-closure; EG, exfoliation; PG, primary open-angle; BCVA, Best-Corrected Visual Acuity; LogMAR, logarithm of the minimum angle of resolution; IOP, intraocular pressure; AL, axial length; HT, hypertension; DM, diabetes mellitus.

**Table 8:** Multivariate analysis of possible associations between FL and demographic parameters.

Parameters	Mini-Cog			G8			ACCI		
	Estimate	95%CI	*p*-Value	Estimate	95%CI	*p*-Value	Estimate	95%CI	*p*-Value
Age, /y	0.11	−0.01, 0.23	0.08	-	-	-	-	-	-
Sex, F/M	0.70	−1.73, 3.13	0.57	0.82	−1.61, 3.25	0.51	0.78	−1.65, 3.21	0.53
Lens, IOL/phakic	−0.18	−2.85, 2.48	0.89	0.72	−1.81, 3.24	0.58	0.36	−2.24, 2.97	0.78
Gla, AC/PG	−3.70	−8.61, 1.21	0.14	−3.67	−8.57, 1.22	0.14	−3.65	−8.55, 1.25	0.14
Gla, EG/PG	0.61	−2.37, 3.60	0.69	1.32	−1.59, 4.24	0.37	1.06	−1.90, 4.02	0.48
BCVA, /LogMAR	5.42	2.28, 8.55	0.0007 **	6.13	3.06, 9.21	<0.0001 **	5.88	2.76, 8.99	0.0002 **
SERE, /D	−0.26	−0.73, 0.21	0.27	−0.25	−0.72, 0.22	0.30	−0.25	−0.73, 0.22	0.29
IOP, /mmHg	−0.03	−0.15, 0.09	0.66	−0.06	−0.18, 0.06	0.34	−0.05	−0.17, 0.07	0.43
AL, /mm	−0.13	−1.07, 0.81	0.79	−0.47	−1.38, 0.45	0.32	−0.33	−1.25, 0.60	0.49
HT, yes/no	−1.64	−4.11, 0.84	0.19	2.11	−0.30, 4.53	0.09	1.99	−0.45, 4.44	0.11
DM, yes/no	−4.47	−7.85, −1.09	0.01 *	4.85	1.49, 8.22	0.005 **	4.7	1.33, 8.07	0.006 **
Mini-Cog	−0.26	−1.53, 1.02	0.69	-	-	-	-	-	-
G8	-	-	-	0.29	−0.39, 0.96	0.40	-	-	-
ACCI	-	-	-	-	-	-	0.31	−0.60, 1.23	0.50

*p*-values are calculated by Generalized Linear Mixed Model. The * and ** denote significant levels of 5% (*p* < 0.05) and 1% (*p* < 0.01), respectively. Mini-Cog, Mini-Cognitive Assessment Instrument; G8, G8 geriatric screening tool; ACCI, Age-Adjusted Charlson Comorbidity Index; 95%CI, 95% confidence interval; IOL, intraocular lens (pseudophakic); Gla, glaucoma; AC, angle-closure; EG, exfoliation; PG, primary open-angle; BCVA, Best-Corrected Visual Acuity; LogMAR, logarithm of the minimum angle of resolution; IOP, intraocular pressure; AL, axial length; HT, hypertension; DM, diabetes mellitus.

**Table 9:** Multivariate analysis of possible associations between FN and demographic parameters.

Parameters	Mini-Cog			G8			ACCI		
	Estimate	95%CI	*p*-Value	Estimate	95%CI	*p*-Value	Estimate	95%CI	*p*-Value
Age, /y	0.09	0.04, 0.14	0.0007 **	-	-	-	-	-	-
Sex, F/M	−0.09	−1.07, 0.90	0.86	−0.15	−1.14, 0.84	0.77	0.02	−0.97, 1.00	0.98
Lens, IOL/phakic	−0.09	−1.21, 1.02	0.87	0.44	−0.62, 1.50	0.42	0.12	−0.98, 1.21	0.84
Gla, AC/PG	−0.90	−2.90, 1.10	0.38	−0.8	−2.80, 1.20	0.43	−0.89	−2.89, 1.10	0.38
Gla, EG/PG	−0.07	−1.33, 1.19	0.91	0.34	−0.89, 1.57	0.59	0.11	−1.14, 1.36	0.86
BCVA, /LogMAR	1.87	0.47, 3.27	0.009 **	2.38	1.01, 3.75	0.0007 **	2.07	0.68, 3.45	0.004 **
SERE, /D	−0.14	−0.34, 0.06	0.18	−0.1	−0.30, 0.10	0.32	−0.15	−0.35, 0.05	0.15
IOP, /mmHg	0.07	0.02, 0.13	0.007 **	0.05	0.00, 0.10	0.049 *	0.06	0.01, 0.12	0.02 *
AL, /mm	−0.21	−0.60, 0.18	0.29	−0.35	−0.74, 0.03	0.07	−0.29	−0.68, 0.10	0.14
HT, yes/no	−0.48	−1.48, 0.53	0.35	0.87	−0.11, 1.86	0.08	0.63	−0.36, 1.62	0.21
DM, yes/no	0.44	−0.92, 1.81	0.52	−0.32	−1.68, 1.05	0.65	−0.33	−1.69, 1.03	0.63
Mini-Cog	−0.15	−0.66, 0.37	0.57	-	-	-	-	-	-
G8	-	-	-	−0.31	−0.58, −0.03	0.03 *	-	-	-
ACCI	-	-	-	-	-	-	0.57	0.20, 0.94	0.003 **

*p*-values are calculated by Generalized Linear Mixed Model. The * and ** denote significant levels of 5% (*p* < 0.05) and 1% (*p* < 0.01), respectively. Mini-Cog, Mini-Cognitive Assessment Instrument; G8, G8 geriatric screening tool; ACCI, Age-Adjusted Charlson Comorbidity Index; 95%CI, 95% confidence interval; IOL, intraocular lens (pseudophakic); Gla, glaucoma; AC, angle-closure; EG, exfoliation; PG, primary open-angle; BCVA, Best-Corrected Visual Acuity; LogMAR, logarithm of the minimum angle of resolution; IOP, intraocular pressure; AL, axial length; HT, hypertension; DM, diabetes mellitus.

**Table 10:** Multivariate analysis of possible associations between FP and demographic parameters.

Parameters	Mini-Cog			G8			ACCI		
	Estimate	95%CI	*p*-Value	Estimate	95%CI	*p*-Value	Estimate	95%CI	*p*-Value
Age, /y	0.01	−0.04, 0.06	0.68	-	-	-	-	-	-
Sex, F/M	1.77	0.80, 2.73	0.0003 **	1.87	0.91, 2.83	0.0001 **	1.77	0.81, 2.73	0.0003 **
Lens, IOL/phakic	−0.14	−1.19, 0.92	0.80	0.05	−0.95, 1.05	0.92	0.04	−0.99, 1.07	0.94
Gla, AC/PG	−2.36	−4.30, −0.41	0.02 *	−2.45	−4.38, −0.51	0.01 *	−2.39	−4.33, −0.45	0.02 *
Gla, EG/PG	0.13	−1.05, 1.31	0.83	0.25	−0.90, 1.40	0.67	0.25	−0.92, 1.42	0.68
BCVA, /LogMAR	−1.27	−2.51, −0.03	0.04 *	−1.21	−2.42, 0.00	0.05	−1.18	−2.41, 0.05	0.06
SERE, /D	−0.17	−0.35, 0.02	0.08	−0.18	−0.36, 0.01	0.06	−0.16	−0.34, 0.03	0.10
IOP, /mmHg	0.03	−0.01, 0.08	0.17	0.03	−0.02, 0.08	0.20	0.03	−0.02, 0.08	0.23
AL, /mm	−0.15	−0.52, 0.23	0.44	−0.22	−0.58, 0.14	0.23	−0.19	−0.56, 0.18	0.31
HT, yes/no	−0.92	−1.90, 0.06	0.07	0.96	0.00, 1.91	0.049 *	1.02	0.05, 1.99	0.04 *
DM, yes/no	−0.04	−1.38, 1.30	0.96	0.13	−1.20, 1.46	0.84	0.07	−1.26, 1.41	0.91
Mini-Cog	0.17	−0.34, 0.67	0.52	-	-	-	-	-	-
G8	-	-	-	0.28	0.01, 0.54	0.04 *	-	-	-
ACCI	-	-	-	-	-	-	−0.14	−0.50, 0.22	0.44

*p*-values are calculated by Generalized Linear Mixed Model. The * and ** denote significant levels of 5% (*p* < 0.05) and 1% (*p* < 0.01), respectively. Mini-Cog, Mini-Cognitive Assessment Instrument; G8, G8 geriatric screening tool; ACCI, Age-Adjusted Charlson Comorbidity Index; 95%CI, 95% confidence interval; IOL, intraocular lens (pseudophakic); Gla, glaucoma; AC, angle-closure; EG, exfoliation; PG, primary open-angle; BCVA, Best-Corrected Visual Acuity; LogMAR, logarithm of the minimum angle of resolution; IOP, intraocular pressure; AL, axial length; HT, hypertension; DM, diabetes mellitus.

## Data Availability

The raw data supporting the conclusions of this article will be made available by the authors on request.

## Abbreviations

The following abbreviations are used in this manuscript:

AC	angle-closure glaucoma
ACCI	Age-Adjusted Charlson Comorbidity Index
AL	axial length
BCVA	best-corrected visual acuity
CGA	comprehensive geriatric assessment
DM	diabetes mellitus
EG	exfoliation glaucoma
FL	fixation loss rate
FN	false-negative rate
FP	false-positive rate
G8	G8 geriatric screening tool
GLMM	generalized linear mixed model
HT	hypertension
IOL	intraocular lens
IOP	intraocular pressure
LogMAR	logarithm of the minimum angle of resolution
MD	mean deviation
PG	primary open-angle glaucoma
PSD	pattern standard deviation
SD	standard deviation
SERE	spherical equivalent refractive error
VF	visual field
